# The role of circulating tumor DNA (ctDNA) in urothelial cancers

**DOI:** 10.37349/etat.2025.1002317

**Published:** 2025-05-19

**Authors:** Jeanny B. Aragon-Ching

**Affiliations:** University of Campania “Luigi Vanvitelli”, Italy; ^1^GU Medical Oncology, Inova Schar Cancer Institute, Fairfax, VA 22031, USA; ^2^Department of Medical Education, University of Virginia, Charlottesville, VA 22904, USA

**Keywords:** Circulating tumor DNA (ctDNA), bladder cancer, urothelial cancer, muscle invasive bladder cancer (MIBC), metastatic urothelial cancer (mUC)

## Abstract

The role of circulating tumor DNA (ctDNA) in urothelial cancers is a rapidly evolving area of research. Urothelial cancer is the most common subtype of bladder cancer, and biomarkers that predict response or prognosticate outcomes have been long sought after. Tumor-informed ctDNA assays have been utilized in several other cancers and increasingly used in both muscle invasive bladder cancer (MIBC) and metastatic urothelial cancer (mUC) to inform treatment decision-making. While a universal consensus on ctDNA testing has not been fully defined and discussed herein, understanding its benefits and limitations is important to help guide the practical application in the clinic.

## Introduction

Bladder cancer was the 7th most common cancer in the world in 2020 and projected to have increasing global burden by 2040 [1]. While other solid tumor cancers have biomarkers in use to help in the diagnosis, predict or prognosticate outcomes, such as the use of prostate specific antigen (PSA) in prostate cancers, urothelial cancer (UC) has no such equivalent markers. There have been huge efforts at identifying biomarkers of response in UC [2], though study results have been largely mixed and inconclusive. Therefore, the use of liquid biopsy and in particular, circulating tumor DNA (ctDNA) arose from the notion that fragments of cell-free DNA (cfDNA) are shed in the blood stream by tumor cells hence are able to be captured by different varying platforms [3]. There are varying roles of obtaining liquid biopsy which can detect minute amounts of disseminated tumor cells [4]. There are distinct advantages in obtaining ctDNA, which includes the ease of technology since it entails obtaining plasma from a blood draw though also wrought with challenges including limitations of yield from a small plasma sample and manner of extraction where the extracted DNA yield may be limited with varying disease volumes [5, 6]. In general, there are two main approaches to detecting ctDNA. One is a tumor-informed platform that involves concurrent sequencing of individual tumors and obtaining plasma from patients to track patient-specific mutations. The other way is to obtain a tumor-agnostic panel with biomarkers that are designed to check for presence of cancers and no correlation to patients’ specific genetic information is needed. While multiple ctDNA studies in other solid tumors exist, particularly for colorectal cancer [7] where decisions regarding omission of adjuvant chemotherapy can be made depending on ctDNA positivity or negativity without detrimental effects to disease-free survival (DFS) or recurrence-free survival (RFS) in stage II colon cancers [8], as well as in lung cancers [9], with generally good negative predictive value for ctDNA negative patients [10], limited data exists in UC. Most of these data are derived from the phase III IMvigor010 trial which was considered a negative adjuvant immune checkpoint inhibitor (ICI) trial to refine the patient population who might benefit from ICI treatment. Regardless, encouraging data is emerging in its utility to inform clinical practice.

## Background on the use of ctDNA and role in UC

Alterations in ctDNA are detectable through polymerase chain reaction (PCR)-based assays or next-generation sequencing (NGS) assays. The ability to detect mutations are measured in ctDNA fractions of 0.01% to 1% and quantifiable as mutant alleles. However, tools that are used to define the tests can bring about false-positive and false-negative results [11]. There are different purposes ranging from detecting minimal residual disease (MRD) in patients undergoing ICIs in bladder cancer, monitoring for recurrence in those who underwent radical surgery, or in earlier disease states such as in non-muscle invasive bladder cancer (NMIBC).

One of the early prognostic trials obtaining ctDNA identified by whole-exome sequencing evaluated 68 patients with locally advanced bladder cancer which showed good prognostic value of ctDNA in accurately identifying patients who developed metastatic relapse with 100% sensitivity and 98% specificity [12]. It also showed the median time to radiographic progression is about 96 days once it is identified to be positive. This was one of the early trials showing monitoring using ctDNA is feasible. A lot of the ctDNA data is derived from the phase III IMvigor010 trial which was one of the first phase III trials that did not meet its primary endpoint of DFS in an adjuvant therapy setting using atezolizumab versus placebo [13]. While the IMvigor010 trial was negative, efforts to define the patient population who would benefit was established with the use of ctDNA. Data surrounding the use of a baseline ctDNA level at C1D1 (cycle 1 day 1) showed the rates of positivity was 214 out of 581 patients (37%) and those who had positive ctDNA results at baseline had an overall worse prognosis [14]. No difference in DFS or overall survival (OS) was seen in those who tested negative for baseline ctDNA. On the other hand, for those who received atezolizumab, testing positive for baseline ctDNA did improve DFS and OS compared to the observation alone arm with a DFS HR (hazard ratio) = 0.58 [95% confidence interval (CI): 0.43–0.79]; *P* = 0.0024, OS HR = 0.59 (95% CI: 0.41–0.86). These results were further confirmed in the updated OS analyses where ctDNA positivity resulted in overall poor OS in the observation arm OS [HR = 6.3 (95% CI: 4.3–9.3)] [15]. However, the degree of ctDNA positivity clearance also dictates improvement in OS outcomes, as depicted by better OS outcomes upon improved clearance of ctDNA with C3D1 of treatment OS of 60 months if 100% clearance was achieved, compared to 34.3 months OS if a 50% to 99% reduction was achieved compared to only 19.9 months of OS if < 50% reduction in ctDNA was achieved. However, all of these were exploratory analyses since the goal of this negative trial is to identify a potential patient population who might benefit from adjuvant atezolizumab. There is no equivalent data arising from another adjuvant ICI trial using nivolumab in the CheckMate 274 trial [16], which is considered a positive trial. Additional studies support the use of ctDNA testing since finding negative ctDNA confers no additional benefit when receiving additional adjuvant therapy [17]. There are several independent retrospective trials that seek to determine the role of ctDNA in different setting other than the laUC [18]. On the other hand, the historical role of the use of ctDNA was in MRD detection in patients with metastatic UC (mUC) [19]. Serial monitoring of ctDNA predicts for disease progression and can allow for dynamic monitoring of changes that help guide and inform treatment [20, 21]. Other potential clinical application in the treatment setting with the use of ICI is early change in the ctDNA clearance, whereby a 3-week increase in the ctDNA level portends a progression-free survival (PFS) with a HR of 7.8 (95% CI 3.1–19.5) and OS of 8 months (95% CI 3.0–21.0) [22]. In addition, obtaining ctDNA in the mUC setting can provide additional information regarding somatic mutational profile in some patients especially with proportions above 2% of plasma cfDNA using whole-exome sequencing and targeted sequencing, where alterations to certain targetable genes (including amplification of *ERBB2* was detected in 20% of patients) [23]. While there are multiple emerging studies regarding benefits of serial ctDNA monitoring in late stages, the role in early diagnostic testing in NMIBC remains limited, with a yield of 50% in those treated with immunotherapy in one small study that included 82 patients [24].

## Future directions

The use of ctDNA is but one way of assessing prognosis but not quite yet predictive of response. Efforts are under way to further improve outcomes by addressing intensification of treatment in several studies. MODERN is an ALLIANCE phase III trial (clinicaltrials.gov NCT05987241) that serves to address the value of intensification of ICI therapy in those who test positive for ctDNA and a way to spare adjuvant therapy to those who might be spared the adverse effects of adjuvant ICI for those who do not need it (see Table 1). Similarly, the IMvigor011 trial (clinicaltrials.gov NCT04660344) will also seek to answer the role of adjuvant atezolizumab in those who are ctDNA positive. On the other hand, this study is also built upon the major role of ctDNA that has not yet been prospectively validated though used retrospectively in major studies. Furthermore, the use of more sensitive or specific assays that combines ctDNA with genetic, epigenetics and a multiomics signature to improve MRD detection would be the way moving forward in improving treatment selection for those who do manifest with positive ctDNA. The inherent challenge is the optimal clinical treatment for those who have positive ctDNA or even rising ctDNA in the absence of objective radiologic imaging findings of metastatic disease. In clinical practice, routine testing for ctDNA is not yet readily adopted. Adaptive clinical trials like MODERN aim to determine if adaptive treatment with ICI or intensified arms can clear ctDNA and potentially lead to a cure or at least improved outcomes. On the other hand, prospective trials to validate ctDNA to ensure it is an appropriate surrogate for prognosticating marker or even in the prediction of response for MRD testing with the use of contemporary agents such as avelumab, enfortumab vedotin, pembrolizumab, would be important to guide decision-making and plans for switching therapies whenever needed. An adaptive biomarker-directed platform study utilizing durvalumab (BISCAY, clinicaltrials.gov NCT02546661) sought to evaluate treatment responses utilizing sequential ctDNA analyses and while PFS results were similar across all combination cohorts [25], changes in the ctDNA and fibroblast growth factor mutations (*FGFR*m) did correlate with outcomes. Beyond obtaining blood samples for ctDNA analyses, other platforms of detection, including obtaining urine samples are also emerging technology [26]. However, despite all the data surrounding its utility in clinical practice, most major associations have not yet actively endorsed obtaining ctDNA as a standard routine practice of biomarker assessment in any stage of UC until further prospective validation studies are performed [27].

**Table 1:** Select studies on UC utilizing ctDNA-guided treatment responses

**Trial name/study cohort**	**Phase of trial/patient population**	***N* of patients**	**Primary endpoint**	**Results/Comments**
IMvigor010	Phase III/ypT2/pT3/pT4 or pN+ or > pT3pN+	500	DFS on adjuvant atezolizumab vs. placebo	Primary endpoint negative DFS atezolizumab vs. placebo; 37% were ctDNA+; ctDNA+ with improved OS in atezolizumab arm (HR 0.59)
Sfakianos et al. [17]	Retrospective/stage I–IV UC	167	Real-world data on DFS with ctDNA, WES, NGS	No DFS benefit from adjuvant atezolizumab if ctDNA– (*P* = 0.34); ctDNA+ shorter DFS (HR = 6.93, *P* < 0.001)
IMvigor011	Phase III/ypT2/pT3/pT4 (if +NAC) or pN+ or > pT3pN+ (if no NAC)	405	DFS for ctDNA+ ≤ 20 weeks after cystectomy	No results yet
MODERN	Phases II and III/ypT2/pT3/pT4 (if +NAC) or pN+ or > pT3pN+ (if no NAC)	1,000	ctDNA clearance proportion; DFS for cohort B and OS for cohort A (phase III nivolumab vs. nivolumab/relatlimab)	No results yet

pT2: pathologic T2 stage; pT3: pathologic T3 stage; DFS: disease-free survival; PFS: progression-free survival; pN+: pathologic node positive; NAC: neoadjuvant chemotherapy; ctDNA: circulating tumor DNA; HR: hazard ratio; NGS: next-generation sequencing; OS: overall survival; UC: urothelial cancer

## Conclusion

In conclusion, the use of ctDNA has emerged as a promising biomarker in the field of UC across disease stages, offering valuable insights into prognosis and potential treatment responses. Despite the challenges associated with ctDNA detection, its utility in clinical practice is becoming increasingly evident. The ability to monitor MRD and predict disease progression through ctDNA analysis provides a dynamic approach to guiding treatment decisions. Future trials, including MODERN or IMvigor011, can further validate the role of ctDNA in treatment intensification for those who require additional treatment or adaptive de-escalation therapy to avert potential toxicity for those who do not require intensive therapy. The next-generation of ctDNA assays aim to further integrate the use of ctDNA with genetic, epigenetic, and multiomics signatures which hold promise for improving treatment selection and outcomes in UC patients. However, prospective validation studies are essential to determine its effectiveness as a routine biomarker and to justify its higher cost compared to standard imaging alone in monitoring disease progression.

## Abbreviations

CI: confidence interval
ctDNA: circulating tumor DNA
DFS: disease-free survival
HR: hazard ratio
ICI: immune checkpoint inhibitor
MIBC: muscle invasive bladder cancer
MRD: minimal residual disease
mUC: metastatic urothelial cancer
OS: overall survival
PFS: progression-free survival
UC: urothelial cancer

## Declarations

### Copyright

© The Author(s) 2025.
